# Modulation of the Vasopressin System in Distributive and Cardiogenic Shock: Theoretical Principles and Practical Applications

**DOI:** 10.3390/jcm15051953

**Published:** 2026-03-04

**Authors:** Alfredo Mauriello, Adriana Correra, Anna Chiara Maratea, Valeria Cetoretta, Francesco Giallauria, Giovanni Esposito, Alfonso Desiderio, Gemma Marrazzo, Biagio Liccardo, Vincenzo Russo, Paolo Trambaiolo, Antonello D’Andrea

**Affiliations:** 1 S.C. Cardiology, Institute National Cancer, IRCCS, Fondazione “G. Pascale”, Via M. Semmola 52, 80131 Naples, Italy; alfredo.mauriello93@libero.it; 2 Cardiology Department, Ospedali Riuniti University Hospital, Viale Pinto 1, 71122 Foggia, Italy; adrianacorrera@gmail.com; 3 Department of Cardiovascular Disease, ASL Napoli 1 Centro, Via Comunale del Principe, 13/a, 80145 Napoli, Italy; annachiara.maratea@gmail.com; 4 Cardiology and Arrhythmology Clinic, Marche Polytechnic University, University Hospital “Ospedali Riuniti”, Via Conca 71, 60126 Ancona, Italy; vcetoretta@gmail.com; 5 Department of Translational Medical Sciences, “Federico II” University of Naples, Via S. Pansini 5, 80131 Naples, Italy; francesco.giallauria@unina.it (F.G.); giovanni.esposito2@unina.it (G.E.); 6 Cardiology and Intensive Care Unit, Department of Cardiology, “Umberto I” Hospital, Via Alfonso De Nicola 1, 84014 Nocera Inferiore, Italy; alf.desiderio@tiscali.it (A.D.); gemmamarrazzo@gmail.com (G.M.); 7 Cardiology Unit, Department of Medical and Translational Sciences, University of Campania “Luigi Vanvitelli”, “V. Monaldi” Hospital, Via Leonardo Bianchi SNC, 80131 Naples, Italy; liccardob@gmail.com (B.L.); vincenzo.russo@unicampania.it (V.R.); 8 Intensive Cardiac Care Unit, Department of Cardiology, Sandro Pertini Hospital, Via Monti Tiburtini, 287, 00157 Rome, Italy; paolo.trambaiolo@gmail.com

**Keywords:** vasopressor, vasopressin, shock, septic shock, intensive cardiac care

## Abstract

Vasodilatory shock, primarily driven by sepsis, remains a leading cause of mortality in intensive care units (ICU), with mortality rates exceeding 90% in refractory cases. While norepinephrine is the first-line vasopressor, prolonged exposure to high doses of catecholamines is linked to severe adverse effects, including myocardial toxicity, arrhythmias, and immunodepression. Consequently, the concept of decatecholaminization, utilizing non-adrenergic vasopressors to reduce catecholamine burden, has emerged as a critical therapeutic strategy. This comprehensive review aims to define the current role of vasopressin and its analogues, terlipressin and selepressin, in managing patients with circulatory shock, evaluating their physiological rationale, clinical benefits, and adverse event profiles. The vasopressin system provides a multimodal approach to hemodynamic stability independent of α-adrenergic stimulation. Arginine vasopressin (AVP) acts on V1a receptors to induce vasoconstriction and improve glomerular filtration, and on V2 receptors for water reabsorption. Clinical trials indicate that while AVP may not reduce overall mortality, it significantly reduces the need for renal replacement therapy (RRT) and offers survival benefits in the less severe shock subgroup. Synthetic analogues like terlipressin offer a longer half-life but carry an increased risk of peripheral ischemia. Conversely, selepressin, a pure V1a agonist, was designed to mitigate fluid retention and edema, though recent trials have not yet demonstrated superior clinical outcomes over placebo. Modulation of the vasopressin system is a cornerstone of decatecholaminization in distributive and cardiogenic shock. Although a universal mortality benefit has not been established, these agents are crucial for protecting renal function, reducing catecholamine toxicity, and lowering the incidence of arrhythmias. Future strategies should focus on precision medicine, utilizing biomarkers like copeptin and artificial intelligence to optimize the timing and selection of multimodal vasopressor therapy.

## 1. Introduction

Vasodilatory shock represents a critical clinical challenge characterized by severe hypotension due to the loss of vascular tone [1,2,3]. Vasodilatory shock is the most common shock syndrome encountered in critically ill patients. Sepsis, its primary cause, accounts for approximately 7% of adult hospital admissions, with an incidence of about 240 cases per 100,000 inhabitants [4]. Septic shock constitutes between 6.8% and 10.4% of intensive care unit (ICU) admissions [5]. Furthermore, approximately 8% of patients hospitalized in the ICU for other reasons develop septic shock during their stay. This condition is the leading cause of death in ICUs. The 28-day mortality is estimated at around 37% in Europe and North America, reaching 45% in specific national contexts such as France [6]. Globally, sepsis-related deaths are estimated at approximately 11 million annually [7]. In cases of shock refractory to conventional therapies, the mortality rate can exceed 90% [8]. Norepinephrine is currently recommended as the first-line vasopressor to restore mean arterial pressure (MAP) [9]. However, exposure to high doses of catecholamines is associated with numerous adverse effects, including direct myocardial toxicity, hyperkinetic cardiac arrhythmias, and immunosuppression [10]. In this context, the concept of “decatecholaminization” has emerged, which involves the use of non-adrenergic vasopressors for the prevention of high-dose catecholamine exposure through early multimodal therapy and improves clinical outcomes [11]. The vasopressin system represents the primary non-adrenergic therapeutic target to counteract refractory vasodilation [12]. Use of vasopressin and its analogues seems to have several advantages in some critical settings. This narrative review aims to define the current role of vasopressin and its analogues in the management of patients with circulatory shock, evaluating both the benefits and potential adverse events.

## 2. Materials and Methods

This article is a narrative review designed to provide a comprehensive synthesis and critical analysis of current knowledge, clinical progress, and future perspectives on the vasopressin system in the context of circulatory shock. Narrative reviews are particularly effective for synthesizing vast and evolving research areas, such as critical care and hemodynamic management.

To identify relevant sources, an extensive literature search was conducted focusing on the core concepts of the title and its related physiological aspects. Following standard practices for scientific reviews, search modalities utilized the biomedical databases PubMed/MEDLINE and EMBASE for the period from January 2015 to January 2026. The search strings and key terms, integrated using Boolean operators (“AND”, “OR”), included combinations of the following concepts:

- Pharmacological Agents: “Vasopressin” (AVP), “Terlipressin”, “Selepressin”, and “Non-adrenergic vasopressors”.
- Clinical Conditions: “Distributive shock”, “Septic shock”, “Cardiogenic shock”, “Vasoplegic syndrome”, and “Refractory hypotension”.
- Therapeutic Themes: “Decatecholaminization”, “Catecholamine-sparing effect”, “Hemodynamic stabilization”, and “V1a receptor selectivity”.
- Diagnosis and Monitoring: “Biomarkers”, “Copeptin”, “Renal replacement therapy” (RRT), and “Ischemic adverse events”.

Document selection focused on English-language articles, including literature reviews, meta-analyses, retrospective and prospective studies, and clinical trials that provided data on the physiology, clinical efficacy, and safety profile of these agents. Although this process does not follow the strict replicability of a systematic review [13], this methodology ensures that the narrative review is evidence-based and covers the major clinical and scientific developments in the field of vasopressin-based therapy.

## 3. Vasopressin and Its Physiology

Vasopressin is synthesized in the hypothalamus. Specifically, its production occurs within the supraoptic and paraventricular nuclei [14]. The hormone is initially synthesized as a protein precursor called a pre-pro-hormone [15]. This precursor travels along the axons of magnocellular neurons, passing through the pituitary stalk to reach the posterior part of the pituitary gland [15].

Vasopressin is then stored in the posterior pituitary gland, also known as the neurohypophysis, predominantly within the intracellular compartment [16]. When the body receives specific stimuli, such as an increase in blood osmolality or a decrease in blood volume, the hormone is released into the systemic circulation [16]. It is important to note that, following stimulation, only a limited portion, approximately 10–20% of the total reserve, can be released immediately into the blood [16].

AVP exerts its pleiotropic effects through the activation of three types of G-protein-coupled receptors:

- V1a: Located on vascular smooth muscle cells, their activation induces vasoconstriction by increasing intracellular calcium levels [17].
- V1b: Situated in the anterior pituitary and the pancreas, they mediate insulin secretion and the stimulation of the corticotropic axis, leading to the release of cortisol [18].
- V2: Located primarily in the renal collecting ducts, they promote water reabsorption through the recruitment of aquaporin-2 channels [18].

Natural vasopressin has a very short plasma half-life, approximately 5–15 min, and lacks receptor selectivity, simultaneously activating all the pathways. Therefore, it influences renal function through two main mechanisms of action, mediated by specific receptors that act on both fluid balance and the organ’s hemodynamics [19].

### 3.1. Regulation of Water Reabsorption

The most well-known effect of vasopressin is its antidiuretic action. The hormone binds to receptors located on the basolateral surface of the renal collecting duct cells. This activation promotes the recruitment of aquaporin-2 channels into the cell membrane, increasing water permeability and allowing for its reabsorption into the bloodstream [20]. In shock situations, however, the activation of these receptors can sometimes be considered problematic, as it could exacerbate oliguria or fluid retention [17].

### 3.2. Improvement of Glomerular Filtration

Unlike norepinephrine, vasopressin has a unique effect on renal hemodynamics. It induces selective vasoconstriction of the glomerular efferent arterioles by acting on receptors, while having a negligible effect on afferent arterioles, as it triggers a local release of nitric oxide that induces vasodilation in this segment. This combination of effects increases the hydrostatic pressure within the glomerulus, promoting an increase in the glomerular filtration rate (GFR) [21,22].

### 3.3. Clinical Effects and Renal Protection in Shock

In states of distributive shock, such as septic shock or vasoplegic shock, the administration of vasopressin or its analogue terlipressin has demonstrated tangible benefits [23,24]. Vasopressin leads to an increase in urine output and creatinine clearance; therefore, it reduces serum creatinine levels compared to treatment with catecholamines alone and ultimately leads to a reduction in the need for renal replacement therapy (RRT) [25]. Kotani et al. [26] in their meta-analysis, including 51 randomized controlled trials totalling 5715 patients, showed that the administration of non-adrenergic agents was associated with a reduced risk of RRT (9 studies; RR, 0.68; 95% CI, 0.57 to 0.81; I^2^ = 0%), especially in patients “at risk” of acute kidney injury.

### 3.4. Central Effect of Vasopressin

In a healthy person, AVP-induced vasoconstriction is often offset by a compensatory decrease in sympathetic heart rate [27]. Interestingly, while AVP is a vasoconstrictor, it often acts to inhibit sympathetic nerve activity centrally to prevent blood pressure from overshooting [28]. AVP acts on the area postrema to inhibit the sympathetic centers in the medulla. AVP enhances the sensitivity of the baroreceptor reflex [28]. When AVP causes blood pressure to rise, it signals the brain to decrease sympathetic outflow more aggressively than other pressors would [28].

In contrast, new analogues such as selepressin or terlipressin have been designed to be pure agonists of specific receptors, seeking to maintain the beneficial pressor and hemodynamic effects without activating the V2 receptors, thereby avoiding forced water reabsorption and the risk of edema [29].

## 4. The Pathophysiology of Shock and the Role of Vasopressin

Shock is a critical clinical condition characterized by inadequate tissue perfusion, leading to cellular hypoxia and organ dysfunction [30]. There are three primary mechanisms for maintaining arterial pressure, each corresponding to specific physiological systems and their related vasopressor agents used in clinical settings [31].

**Figure 1:**
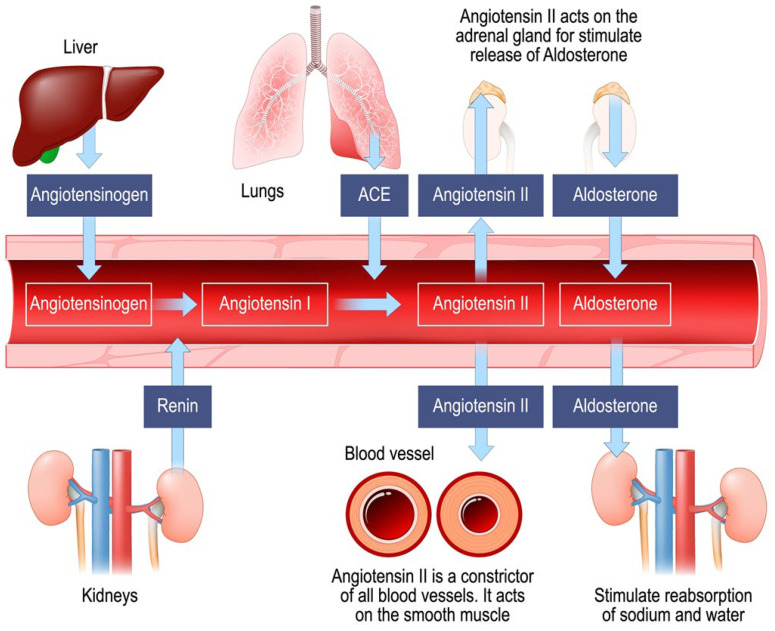
Schematic representation of the renin–angiotensin–aldosterone system (RAAS); ACE: angiotensin-converting enzyme.

1. The adrenergic sympathetic nervous system [31]: this system acts primarily through the release of catecholamines. Norepinephrine is considered the first-line vasopressor for the treatment of septic shock. It exerts its action by binding to α-adrenergic receptors, causing arterial and venous vasoconstriction, and to β-adrenergic receptors, exerting a positive inotropic effect on the heart [32]. However, excessive exposure to catecholamines can cause adverse effects such as arrhythmias, myocardial toxicity, and immunosuppression.
2. The vasopressin system [33]: this mechanism is independent of α-adrenergic stimulation, which makes vasopressin particularly useful in shock states where adrenergic receptors are down-regulated or less sensitive. In addition to its pressor effect, the system regulates water reabsorption in the kidneys.
3. The renin–angiotensin–aldosterone system (RAAS) [34]: this system produces Angiotensin II, a potent synthetic vasoconstrictor approved for clinical use in the treatment of vasodilatory shock. Angiotensin II acts by binding to type 1 (AT1) receptors present in the blood vessels, kidneys, brain, and heart. In addition to causing potent venous and arterial vasoconstriction to restore pressure, this system regulates aldosterone synthesis and fluid-electrolyte balance. Figure 1 represents the renin–angiotensin–aldosterone system (RAAS).

**Figure 2:**
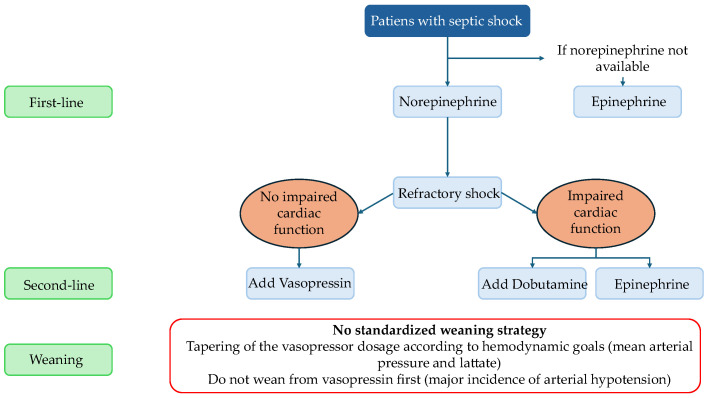
Flow chart of management of vasopressors in patients with septic shock.

There is no universal consensus definition of refractory shock. Proposed definitions include failure to achieve a BP goal despite vasopressor therapy, need for rescue vasopressor therapy, or need for high vasopressor doses [35]. In situations of refractory shock, the use of vasopressors that simultaneously target these three different systems, implementing a multimodal therapy, can help restore hemodynamic stability while reducing the toxic load of individual substances, a concept known as “decatecholaminization” [36]. Figure 2 represents the flow chart of management of vasopressors in septic shock.

In septic shock and other forms of distributive shock, the vasopressin system undergoes biphasic alterations. In the initial phase, plasma AVP levels increase significantly as a compensatory mechanism. However, as shock progresses, a relative vasopressin deficiency is observed in up to one-third of patients, due to the depletion of neurohypophyseal stores and the impairment of baroreceptive reflexes. Unlike healthy subjects, patients in septic shock show marked hypersensitivity to exogenous vasopressin. In contrast, in cardiogenic shock, vasopressin levels are generally higher than in septic shock, reflecting a different neuroendocrine response to low cardiac output [37]. The administration of AVP in these states aims to restore vascular tone through a pathway independent of adrenergic receptors, which are often down-regulated during sepsis [36,38].

Decatecholaminization is a clinical concept that consists of limiting the use of adrenergic vasopressors, such as norepinephrine, in favor of non-adrenergic agents to reduce the so-called “vasopressor load” [39]. This strategy is considered one of the primary goals of using adjunctive vasopressors in septic shock [40]. The theoretical and practical foundation of this approach is based on the following points:

Reduction in toxicity: Excessive or prolonged exposure to high doses of catecholamines is associated with severe adverse effects, including direct myocardial toxicity through increased inflammation and necrosis, hyperkinetic cardiac arrhythmias, oxidative stress, and immunosuppression [40,41].

Overcoming receptor resistance: In severe septic shock, down-regulation of α-adrenergic receptors often occurs, making patients less responsive to norepinephrine; adding drugs that act on independent pathways, such as vasopressin or Angiotensin II, allows for more efficient restoration of vascular tone [42].

Sparing effect: The use of vasopressin or its analogues has demonstrated a consistent ability to reduce norepinephrine requirements, allowing the target blood pressure to be maintained with less dangerous adrenergic doses [42].

Immune preservation: Unlike norepinephrine, which can contribute to the immunoparalysis typical of sepsis, vasopressin does not appear to negatively alter cytokine concentrations or compromise host defenses [43].

In summary, decatecholaminization aims to shift from a single-drug approach at massive doses to early multimodal therapy, targeting different physiological systems simultaneously to improve clinical outcomes and reduce complications related to adrenergic stress.

## 5. Vasopressin and Its Analogues in Various Types of Shock

The pharmacokinetic differences between AVP and its synthetic analogues, terlipressin and selepressin, primarily concern their half-life, duration of action, and metabolic pathways [36].

### 5.1. Arginine Vasopressin (AVP)

Natural arginine vasopressin is characterized by an extremely rapid kinetic profile. Its half-life is very short, estimated between 5 and 15 min, although some sources indicate approximately 6 min or a range of 10–35 min. The biological effect persists for only 30–60 min. It is rapidly excreted and degraded by renal and hepatic vasopressinases. The standard dosage is 0.03 IU/min, with a titration range starting from 0.01 IU/min, which can be increased every 10–15 min, generally up to 0.03–0.04 IU/min [36].

### 5.2. Terlipressin

Terlipressin is a synthetic analogue designed for prolonged stability. It acts as a prodrug that is gradually cleaved into active vasopressin by endothelial peptidases. It has a significantly longer half-life compared to the natural hormone, reaching up to 6 h. Consequently, its clinical effects last between 2 and 10 h. The initial dosage is 1.3 µg/kg/h or a fixed dose of 2 mg/24 h, up to 4–6 mg/24 h [36].

### 5.3. Selepressin

Selepressin exhibits intermediate pharmacokinetic characteristics. In patients with septic shock, the terminal half-life is approximately 2.5 h, whereas in healthy subjects, it is 1.5 h. This difference is due to a 30% reduction in clearance in critically ill patients, likely resulting from impaired renal function. It possesses a very rapid initial distribution and elimination phase of about 10 min. It has a steady-state volume of distribution between 18 and 31 L, indicating significant extravascular distribution. The time required to reach steady-state plasma concentrations is approximately 7 h. The dosage range is between 0.17 and 1.33 µg/kg/h [36]. Table 1 summarizes the pharmacokinetic parameters of vasopressin and its analogues.

Conventional methods of comparing total vasopressor dose among patients include conversion to norepinephrine equivalents [9]. Table 2 summarizes the conversion to norepinephrine equivalents [44].

Actually, norepinephrine is the most used vasopressor in vasoplegic shock, because, compared to other vasopressors, it has highly predictable and potent efficacy, a lower risk of arrhythmias than epinephrine and does not significantly raise lactate levels.

## 6. Use of Vasopressin and Analogues in Shock

The clinical application of vasopressin and its analogues varies significantly depending on the etiology of the shock.

### 6.1. Septic Shock

Guidelines suggest adding vasopressin as a second-line agent when norepinephrine doses exceed 0.25–0.50 µg/kg/min [36].

#### 6.1.1. Arginine Vasopressin

Large studies such as Vasopressin and Septic Shock Trial (VASST) [45] and VAsopressin versus Noradrenaline as Initial therapy in Septic sHock (VANISH) [46] have confirmed the norepinephrine-sparing effect but have not demonstrated a reduction in overall mortality, although benefits were observed in less severe subgroups or when combined with corticosteroids. In the VASST [45], 778 patients with septic shock (mean arterial pressure (MAP) < 65 mmHg receiving > 5 µg/kg/min of norepinephrine) were randomized to receive either norepinephrine alone or norepinephrine plus low-dose vasopressin (0.01 to 0.03 U/min). While no survival benefit was demonstrated in the whole population, a trend towards reduced 28-day mortality (26.5% vs. 35.7%, *p* = 0.05) and significantly lower 90-day mortality (35.8% vs. 46.1%, *p* = 0.04) was observed in less severe patients, requiring norepinephrine doses < 15 µg/min to maintain MAP > 65 mmHg.

A post hoc analysis of the VASST also demonstrated a significant reduction in 28-day and 90-day mortality rates with vasopressin administration compared to norepinephrine administration in patients with blood lactate concentration ≤ 2 mmol/L [47]. In the VANISH trial [46], 421 patients with septic shock were randomized to receive as first-line vasopressor either norepinephrine or vasopressin (titrated on MAP level up to 0.06 U/min). There was no significant difference in mortality rates between groups. However, a post hoc analysis of the VASST and the results of the VANISH trial (25.4% vs. 35.3%, *p* < 0.007) showed that vasopressin was associated with a reduction in the need for RRT [9]. Furthermore, the immunological effect of vasopressin was suggested in a post hoc analysis of the VANISH trial, which found that the anti-cytokine effect of hydrocortisone was enhanced when combined with vasopressin [48].

#### 6.1.2. Terlipressin

This analog has greater selectivity for V1a receptors and a longer half-life (approximately 6 h). In a meta-analysis [49] including 9 studies with 850 participants, the use of terlipressin was associated with a reduction in mortality in patients under 60 years of age (relative risk (RR), 0.66 [0.50 to 0.86]; *p* = 0.002), but it carries a significantly higher risk of peripheral ischemia (Odds Ratio (OR), 8.65 [1.48 to 50.59]; *p* = 0.02). Overall, no significant difference in mortality was observed between the terlipressin and catecholamine groups (RR, 0.85 [0.70 to 1.03]; *p* = 0.09). It has been suggested that microcirculatory flow is a strong predictor of clinical outcome in shock states; in elderly patients, terlipressin may not effectively improve microcirculation, explaining the lack of mortality reduction in this age group. Additionally, it has been associated with a higher incidence of acute pulmonary edema and diarrhea [50].

#### 6.1.3. Selepressin

A pure V1a agonist designed to reduce pulmonary edema and capillary leak without V2-mediated effects. Despite promising premises, the Selepressin Evaluation Programme for Sepsis-induced Shock-Adaptive Clinical Trial (SEPSIS-ACT) study [51] did not show superior benefits over placebo in terms of ventilator- or vasopressor-free days. This adaptive phase 2b/3 trial (868 patients) was stopped for futility, with no significant differences in 90-day mortality or RRT-free days. It may be considered when the risk of acute pulmonary edema is high, although its superiority in terms of mortality remains unproven.

### 6.2. Vasoplegic Shock or Post-Cardiac Surgery

The use of AVP is associated with better hemodynamic stability and a lower incidence of post-operative tachyarrhythmias compared to norepinephrine [52]. In the Vasopressin Versus Norepinephrine for the Management of Septic Shock in Cancer Patients (VANCS) trial, a double-blind trial [53] of 330 patients, the primary endpoint (a composite of mortality or severe complications) occurred in 32% of the vasopressin group versus 49% of the norepinephrine group (Hazard Ratio (HR), 0.55; 95% confidence interval (CI), 0.38 to 0.80; *p* = 0.0014). The vasopressin group also showed a lower occurrence of atrial fibrillation (63.8% vs. 82.1%; *p* = 0.0004).

### 6.3. Hemorrhagic Shock

Low-dose vasopressin supplementation has been shown to significantly reduce the need for blood product transfusions and the risk of deep vein thrombosis. In a randomized trial [54] of 100 trauma patients, those receiving AVP required significantly less blood products at 48 h (median 1.4 L vs. 2.9 L; *p* = 0.01) and had fewer cases of deep vein thrombosis (11% vs. 34%; *p* = 0.02).

### 6.4. Cardiogenic Shock

In cases of cardiogenic shock with hypotension, early administration of vasopressors such as norepinephrine is recommended to preserve perfusion in critical organs (European Society of Cardiology class IIb/B) [55]. In other hand, the administration of vasopressors to augment perfusion is associated with an increase in LV afterload, negatively affecting myocardial contractility. Therefore, guidelines suggest the use of a combination with an inotrope (American College of Cardiology class I/B-NR, European Society of Cardiology class IIb) [55,56].

The utility of a catecholamine-sparing vasopressor strategy using vasopressin has not been widely examined in cardiogenic shock [57,58]. However, the use of adrenergic vasopressor was associated with several adverse effects, including tachycardia, increased myocardial oxygen consumption and pulmonary vasoconstriction. Therefore, some recent studies have evaluated the safety and efficacy of vasopressin and analogues in cardiogenic shock. Sarma et al. [59] in a retrospective study including 721 patients with cardiogenic shock showed that vasopressin was associated with lower in-hospital mortality (adjusted OR 0.59, 95% CI, 0.35–0.99, *p* = 0.05). Vasopressin use was also associated with lower mortality in the high-dose vasopressors defined as ≥0.3 mcg/kg/min of norepinephrine equivalent group (unadjusted OR 0.54, 95% CI, 0.32–0.92, *p* = 0.02). Nguyen et al. [60], in their retrospective study including 100 patients with cardiogenic shock (76 males, median age was 64 years) showed that vasopressin was effective in increasing MAP and reducing norepinephrine requirements in 55% of patients. Although the initial pressure response was not directly associated with 30-day survival, surviving patients exhibited a significantly higher MAP and lower norepinephrine doses starting from the fourth hour of treatment (*p* = 0.32).

In conclusion, vasopressin appears to have the greatest effect in patients with cardiogenic shock who are oliguric, due to its impact on renal function and low systemic vascular resistance. Furthermore, there are emerging data indicating that vasopressin, in this setting, compared with catecholamines, may result in selective vasoconstriction in the systemic circulation and cause pulmonary arterial vasodilation, thereby reducing right ventricular afterload [61]. Therefore, the use of vasopressin is useful in vasoplegic-CS phenotype, right ventricle failure with pulmonary vasoconstriction concerns, and arrhythmia-prone patients, whereas its use may be dangerous in severe LV failure with afterload sensitivity.

However, other studies, such as RCTs, to confirm the role of vasopressin in cardiogenic shock are needed.

## 7. Side Effects of Vasopressin and Its Analogues Use

The infusion of vasopressin and its analogues, while effective in reducing catecholamine requirements, is associated with several side effects, the most relevant of which are ischemic, cardiac, and metabolic effects. The primary adverse effects reported in the literature consist of ischemic [49,62,63], hemodynamic [64,65], and renal complications [66].

### 7.1. Ischemic Complications

This is the most frequent and feared side effect, due to the potent vasoconstrictive action mediated by receptors that can lead to peripheral and digital ischemia. This manifests as cyanosis or necrosis of the extremities [62]. The incidence of digital ischemia is significantly higher with the use of analogues such as terlipressin compared to norepinephrine (OR, 8.65(1.48 to 50.59); *p* = 0.02) [49]. Ischemia can also develop at the skin level; ischemic lesions can affect up to one-third of patients exposed to vasopressin, with risk factors including overweight or the concomitant use of high doses of norepinephrine [63]. Finally, mesenteric and myocardial ischemic lesions may occur [63].

### 7.2. Hemodynamic and Cardiac Effects

As a pure vasoconstrictor, vasopressin markedly increases left ventricular afterload, which can cause a decrease in cardiac output due to ventricular-arterial uncoupling, especially in patients with already compromised systolic function [64]. Alongside hemodynamic effects, arrhythmic effects may occur [65]. Bradycardia is a common effect that can be induced by both vasopressin and terlipressin, but arrhythmias such as atrial fibrillation occur only rarely (OR 0.77 (0.67–0.88), *p* < 0.001) [65].

### 7.3. Metabolic and Renal Effects

Receptor stimulation promotes the reabsorption of free water, which can dilute blood sodium levels, causing hyponatremia, as well as fluid retention and the potential exacerbation of oliguria [66]. Diarrhea is particularly reported with the use of terlipressin [51].

### 7.4. Coagulation and Other Effects

Vasopressin stimulates the release of von Willebrand factor and platelet activation, potentially promoting a hypercoagulable state [67]. If the vasopressin infusion is stopped abruptly or before the discontinuation of norepinephrine, the patient may experience a rapid drop in blood pressure [66].

## 8. Practical Recommendations for Clinical Use

The integration of vasopressin and its analogues into clinical practice requires precise management to maximize the “decatecholaminization” effect while minimizing high-dose catecholamine exposure risks.

### 8.1. Initiation Timing and Dosage

Guidelines suggest adding vasopressin as a second-line agent when norepinephrine doses exceed 0.25–0.50 µg/kg/min to maintain the target MAP. For AVP, the infusion dosage should generally be maintained at 0.03 IU/min. While titration can start from 0.01 IU/min, doses should generally not exceed 0.03–0.04 IU/min, as higher doses are associated with risks such as myocardial or mesenteric ischemia without clear additional benefits. In patients with less severe shock, such as norepinephrine < 15 µg/min or with blood lactate concentrations ≤ 2 mmol/L, the early administration of vasopressin may offer survival benefits.

### 8.2. Safety Monitoring

Continuous monitoring of extremity perfusion is essential, especially in overweight patients or those receiving high doses of norepinephrine, to early detect signs of digital cyanosis or necrosis. In septic shock, vasopressin may be preferred to reduce the need for RRT, as it increases glomerular hydrostatic pressure and GFR. Due to V2 receptor activation, clinicians must monitor dilutional hyponatremia and fluid retention. Pure V1a agonists like selepressin were designed to mitigate the risk of pulmonary edema and fluid overload, though current clinical evidence has not yet demonstrated their superiority in terms of mortality.

### 8.3. Weaning Strategy

During the resolution phase of shock, vasopressin should not be the first vasopressor to be discontinued. Abruptly stopping vasopressin before norepinephrine is associated with a higher incidence of arterial hypotension, likely due to the relative vasopressin deficiency and neurohypophysis store depletion observed during shock progression.

## 9. Future Perspectives

Future research is shifting toward precision medicine to identify patients who would benefit most from the vasopressin system. The use of biomarkers such as copeptin or dynamic osmotic stimulation tests could assist in the early diagnosis of AVP deficiency. Copeptin is considered the most reliable surrogate biomarker for assessing vasopressin levels. Because natural vasopressin has a very short half-life and is unstable, copeptin, which is secreted in equimolar amounts by the neurohypophysis, is easier to measure in a clinical setting. The use of dynamic tests, such as hypertonic saline infusion to stimulate copeptin release and accurately diagnose vasopressin axis deficiency, has been proposed. Furthermore, the integration of artificial intelligence and reinforcement learning algorithms could optimize the exact timing of therapy initiation based on “vasopressor load” rather than just the norepinephrine dose, as well as weaning strategies. Another area of interest is early multimodal therapy, which combines low doses of different vasopressors (AVP, angiotensin II, norepinephrine) to simultaneously target different physiological systems. In the future, evaluating the genetic variability of vasopressin or adrenergic receptors may allow for the personalization of vasopressor therapy based on the individual patient’s biological profile.

## 10. Conclusions

The modulation of the vasopressin system remains a fundamental pillar in the management of distributive and cardiogenic shocks. Although AVP and its analogues have not yet demonstrated a clear, universal benefit on global mortality, their role in decatecholaminization is crucial for mitigating the cardiotoxicity associated with catecholamines. The preservation of renal function and the reduction in arrhythmias represent significant clinical benefits. Vasopressin serves as an adjunct in various shock states, septic, vasoplegic, cardiogenic with oliguria or right ventricular failure, and hemorrhagic, by enhancing hemodynamic stability, reducing catecholamine requirements, and protecting organ function, particularly through its renal-sparing and anti-arrhythmic effects. However, clinical use must be carefully balanced against the risk of peripheral and digital ischemic events, especially when using long-acting analogues

## Figures and Tables

**Table 1:** Pharmacokinetic parameters of vasopressin and its analogues.

Parameter	Vasopressin	Terlipressin	Selepressin
Half-life	5–15 min	~6 h	~2.5 h (critically ill)
Duration of action	30–60 min	2–10 h	Intermediate
Metabolism	Hepatic/renal vasopressinases	Cleavage by peptidase (prodrug)	Peptidase degradation and renal excretion

**Table 2:** Conversion between several vasopressors and norepinephrine equivalents [44].

Drug	Dose	Norepinephrine Equivalent
Epinephrine	0.1 μg/kg/min	0.1 μg/kg/min
Dopamine	15 μg/kg/min	0.1 μg/kg/min
Norepinephrine	0.1 μg/kg/min	0.1 μg/kg/min
Phenylephrine	1 μg/kg/min	0.1 μg/kg/min
Vasopressin	0.04 U/min	0.1 μg/kg/min

## Data Availability

No new data were created or analyzed in this study.
